# Discordant Phylogenomic Placement of Hydnoraceae and Lactoridaceae Within Piperales Using Data From All Three Genomes

**DOI:** 10.3389/fpls.2021.642598

**Published:** 2021-04-12

**Authors:** Matthias Jost, Marie-Stéphanie Samain, Isabel Marques, Sean W. Graham, Stefan Wanke

**Affiliations:** ^1^Institut für Botanik, Technische Universität Dresden, Dresden, Germany; ^2^Instituto de Ecología, A.C., Red de Diversidad Biológica del Occidente Mexicano, Pátzcuaro, Mexico; ^3^Department of Botany, University of British Columbia, Vancouver, BC, Canada; ^4^Plant-Environment Interactions and Biodiversity Lab, Forest Research Centre, Instituto Superior de Agronomia, Universidadede Lisboa, Lisbon, Portugal

**Keywords:** Aristolochiaceae, *Hydnora*, *Prosopanche*, *Lactoris*, *Verhuellia*, plastome, mitochondrial, nuclear

## Abstract

Phylogenetic relationships within the magnoliid order Piperales have been studied extensively, yet the relationships of the monotypic family Lactoridaceae and the holoparasitic Hydnoraceae to the remainder of the order remain a matter of debate. Since the first confident molecular phylogenetic placement of Hydnoraceae among Piperales, different studies have recovered various contradictory topologies. Most phylogenetic hypotheses were inferred using only a few loci and have had incomplete taxon sampling at the genus level. Based on these results and an online survey of taxonomic opinion, the Angiosperm Phylogeny Group lumped both Hydnoraceae and Lactoridaceae in Aristolochiaceae; however, the latter family continues to have unclear relationships to the aforementioned taxa. Here we present extensive phylogenomic tree reconstructions based on up to 137 loci from all three subcellular genomes for all genera of Piperales. We infer relationships based on a variety of phylogenetic methods, explore instances of phylogenomic discordance between the subcellular genomes, and test alternative topologies. Consistent with these phylogenomic results and a consideration of the principles of phylogenetic classification, we propose to exclude Hydnoraceae and Lactoridaceae from the broad circumscription of Aristolochiaceae, and instead favor recognition of four monophyletic and morphologically well circumscribed families in the perianth-bearing Piperales: Aristolochiaceae, Asaraceae, Hydnoraceae, and Lactoridaceae, with a total of six families in the order.

## Introduction

The magnoliid clade Piperales represents the largest angiosperm order outside the eudicots and monocots, as it includes some 4,200 species in 16 genera (Meng et al., 2002; Quijano-Abril et al., 2006; Wanke et al., 2006; Oelschlägel et al., 2011; Wagner et al., 2012; Frenzke et al., 2015; Sinn et al., 2015; Bolin et al., 2018; Funez et al., 2019). Members of this major angiosperm lineage, with an estimated crown diversification of (174-)148(-124) Myr (Salomo et al., 2017) have a nearly worldwide distribution and are present in most terrestrial biomes, occurring from sea level to high mountain areas above the tree line. The order is the most morphologically diverse magnoliid lineage (Isnard et al., 2012), comprising nearly all growth and life forms, including geophytes, epiphytes, annuals, perennials, herbs, succulents, shrubs, trees, lianas, aquatic plants, and parasites (Wanke et al., 2007a; Isnard et al., 2012). In addition, their floral morphology is extremely diverse, ranging from reduced perianth-less, and likely wind-pollinated flowers in Piperaceae and Saururaceae, to insect-trapping flowers in, for example, Aristolochiaceae, and extremely modified (and at least partially subterranean) beetle-pollinated flowers in Hydnoraceae (Bolin et al., 2009; Oelschlägel et al., 2009, 2015; Seymour et al., 2009). Piperales have been the subject of extensive studies in a broad range of scientific fields, including pharmacological investigations of *Aristolochia* (Sati et al., 2011), *Piper* (Zaveri et al., 2010; Ahmad et al., 2012; Shah et al., 2016), *Peperomia* (Hamid et al., 2007), and *Thottea* (Raju and Ramesh, 2012), and investigations into the repelling properties of essential oils of certain *Piper* species to fire ants (Souto et al., 2012), the cattle tick (Silva et al., 2009), and other arthropods (Mamood et al., 2017). Other studies have focused on their conservation biology (Stuessy et al., 1992; Ricci, 2001), pollination biology (Oelschlägel et al., 2016), floral development (Jaramillo et al., 2004; Samain et al., 2010; Pabón-Mora et al., 2015, 2020), the evolution of epiphytism and fruit traits (Frenzke et al., 2016), and ecological interactions between *Piper* and ants (Wisniewski et al., 2019). A recent study on Aristolochiaceae and other host plants of butterflies (Allio et al., 2021) suggests that the evolutionary success of insects may be linked to recurrent changes in host plants (food sources); these changes have left traces of genetic adaptations in their genomes and are also associated with accelerated diversification. From a morphological point of view, Lactoridaceae, endemic to the Juan Fernández Islands, are unique in angiosperms for their saccate pollen (Zavada and Taylor, 1986). Also unique are the Hydnoraceae, to date the only confirmed family of holoparasitic plants outside the eudicot and monocot radiation, whose type genus was first described as a fungus (Thunberg, 1775). Their extremely modified morphology, including the complete absence of leaves, led Musselman and Visser (1986) to suggest that *Hydnora* is the strangest plant in the world (Thorogood, 2019).

**FIGURE 1:**
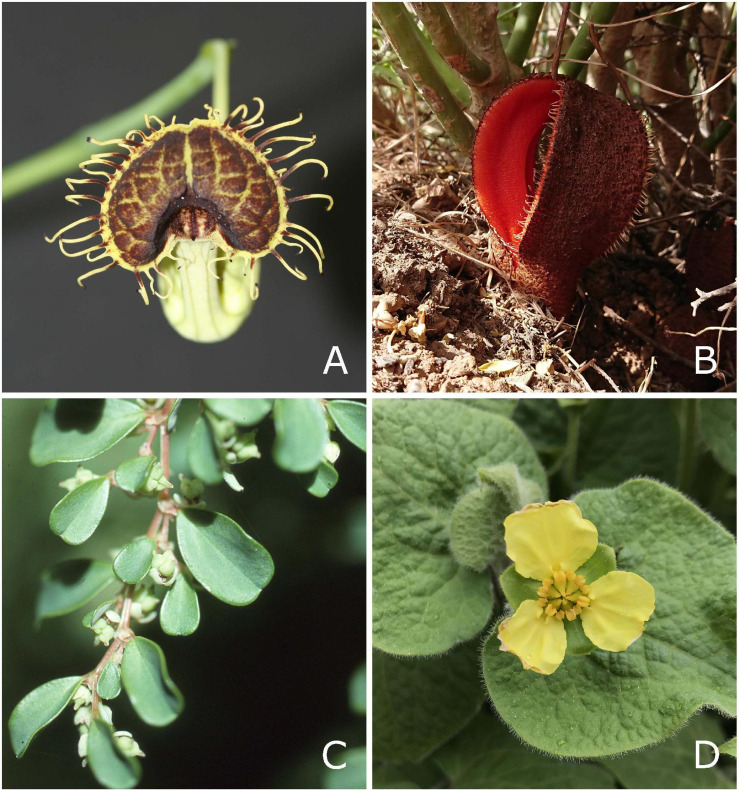
Representatives of perianth-bearing Piperales. **(A)** Flower of *Aristolochia fimbriata* (Aristolochiaceae), **(B)** flower of *Hydnora africana* (Hydnoraceae), **(C)** *Lactoris fernandeziana* with fruits (Lactoridaceae, provided by Tod Stuessy), and **(D)** flower of *Saruma henryi* (Asaraceae, provided by Christoph Neinhuis).

Following the Piperales classification used by Horner et al. (2015), who recognized six families with distinctive morphology, all of which previous studies had recovered as monophyletic, the order consists of: Piperaceae (*Piper*, *Peperomia*, *Manekia*, *Verhuellia*, and *Zippelia*), Saururaceae (*Anemopsis*, *Gymnotheca*, *Houttuynia*, and *Saururus*), Asaraceae (*Asarum*, *Saruma*), Lactoridaceae (*Lactoris*), Hydnoraceae (*Hydnora*, *Prosopanche*), and Aristolochiaceae (*Aristolochia*, *Thottea*). The former two families are the perianth-less Piperales and the latter four are the perianth-bearing members of the order (Figure 1). Relationships at the genus level within Piperaceae and Saururaceae are generally well resolved (Meng et al., 2002; Jaramillo et al., 2004; Wanke et al., 2007b; Massoni et al., 2014), unlike those within the perianth-bearing clade. All six family names were validly published in the 18th and 19th centuries, the youngest one more than 130 years ago, and so they have been accepted as well-defined families for a long time (Jussieu, 1789; Giseke, 1792; Ventenat, 1799; Agardh, 1821; Lestiboudois, 1826; Engler, 1887), with generally few changes of taxonomic rank.

Results of molecular phylogenetic analyses of Piperales in previous studies are generally congruent with respect to the placement of Lactoridaceae. Ignoring the placement of Hydnoraceae, Lactoridaceae are typically recovered as the sister group of Aristolochiaceae, including the studies by Soltis et al. (2000) (based on one mitochondrial and two plastid loci, *Thottea* not included), Qiu et al. (2000) (one nuclear, two mitochondrial, and two plastid loci), Neinhuis et al. (2005) (one plastid locus), Wanke et al. (2007b) (two plastid loci), Wanke et al. (2007a) (one plastid locus), and Massoni et al. (2014) (two nuclear, four mitochondrial, and six plastid loci), and all with poor to moderate support, and with Asaraceae then recovered as the sister group to this clade. However, Jaramillo et al. (2004) recovered a poorly supported clade of Lactoridaceae and Asaraceae, with Aristolochiaceae sister to this clade (one nuclear and two plastid loci), although *Thottea* was missing in their sampling.

Studies that included the holoparasitic Hydnoraceae led to the recovery of multiple different topologies within the perianth-bearing Piperales (Nickrent et al., 2002; Naumann et al., 2013; Massoni et al., 2014). For example, a five-gene analysis (one nuclear, two mitochondrial and two plastid loci) by Nickrent et al. (2002) recovered Hydnoraceae within the clade of perianth-bearing Piperales, although with poor support, and the whole clade as a polytomy comprising *Aristolochia*, *Lactoris*, a clade of *Asarum* and *Saruma*, as well as a clade of *Hydnora* and *Prosopanche* (the two genera in Hydnoraceae). A six-gene analysis (two nuclear and four plastid loci) in the same study placed Lactoridaceae as the sister group of Hydnoraceae, with Aristolochiaceae then sister to this clade, again with poor support (*Thottea* and Asaraceae were not included in the sampling). Note that in the study by Nickrent et al. (2002), the sampled plastid genes in that study were coded as missing for Hydnoraceae and were later shown to be missing from their plastomes (Naumann et al., 2016; Jost et al., 2020). Naumann et al. (2013) recovered Hydnoraceae as the sister group of Aristolochiaceae from analysis of their 19-gene matrix (14 nuclear, two mitochondrial, and three plastid loci), of which 16 loci are present in Hydnoraceae (although none of the plastid genes). The latter topology had moderate support, with Lactoridaceae sister to the clade comprising Hydnoraceae and Aristolochiaceae. A study that examined 12 loci (two nuclear, four mitochondrial, and six plastid loci) (Massoni et al., 2014) instead recovered Hydnoraceae as the sister group of a clade comprising Lactoridaceae and Aristolochiaceae. In that study, the placement of *Lactoris* as the sister group of *Aristolochia* and *Thottea* received poor support in the maximum likelihood (ML) analysis, as did the sister relationship of *Hydnora* to this clade. *Prosopanche* was not included in their study. The very short estimated branches separating the families in perianth-bearing Piperales are noticeable, and are in close proximity to the extremely long branch leading to *Hydnora*. To date, there has been no phylogenetic study that includes all genera of Piperales.

Apart from these uncertainties on the relationships within the order, the composition of Piperales in terms of its constituent families has also fluctuated in recent angiosperm-wide classification schemes. For example, Angiosperm Phylogeny Group (APG) et al. (2016) accepted only three families in Piperales (Aristolochiaceae, Piperaceae, and Saururaceae), as they decided to lump the families Hydnoraceae and Lactoridaceae with Aristolochiaceae; however, all three families had been recognized in previous iterations of the angiosperm system (APG, 1998, 2003, 2009). APG IV made this decision based on a survey to experts in angiosperm taxonomy addressing various aspects of classification (Christenhusz et al., 2015). However, the question posed to taxonomic experts focused heavily on the position of *Lactoris* in the order, without consideration of Hydnoraceae. Only a single expert noted the phylogenetic evidence on the placement of Hydnoraceae at that time. Despite this, Christenhusz et al. (2015) argued that this did not matter, as Hydnoraceae might also be nested in Aristolochiaceae, and so proposed that it should comprise four subfamilies (i.e., Asaroideae, Hydnoroideae, Aristolochioideae, and the newly proposed Lactoridoideae).

At the time of the survey only three studies had sufficiently sampled the aforementioned families, and each recovered contradictory and poorly supported topologies concerning their interrelationships (Nickrent et al., 2002; Naumann et al., 2013; Massoni et al., 2014). Even ignoring the placement of Hydnoraceae, almost half of the respondents did not favor a three-family system for the order (i.e., ∼46% of experts who voiced their opinion were split between two alternative fragmentations of Aristolochiaceae, biasing the answer to the simpler system). For these reasons, we argue that the suggestions made by Christenhusz et al. (2015) and their implementation in and their implementation in and their implementation in APG (2016) potentially problematic and warrant reconsideration.

Prior to the inclusion of Lactoridaceae and Hydnoraceae in Aristolochiaceae, various studies based on molecular data reported Aristolochiaceae as non-monophyletic, with Lactoridaceae depicted as the sister group of subfamily Aristolochioideae (Qiu et al., 2000; Soltis et al., 2000; Neinhuis et al., 2005; Wanke et al., 2007a,b). In contrast, inferences based on morphological data supported the monophyly of Aristolochiaceae, but were ambiguous about the placement of *Lactoris* (Kelly and González, 2003). The two subfamilies Aristolochioideae and Asaroideae were each recovered as monophyletic in all of these studies. When one traditionally recognized family is placed within another in phylogenetic analyses, Smith et al. (2006) lay out three different options: (1) recognition of the paraphyletic taxon; (2) splitting up the larger family into one or more smaller ones; and (3) lumping the paraphyly-causing family into the family it is nested within. Most systematists, including us, would consider the first option undesirable, but several criteria can be used to decide between the latter two.

One consideration when deciding whether to lump a particular family into another is whether monotypic families should be avoided or not. According to Backlund and Bremer (1998), there is no definitive answer to this question, and arguments for both points of view have to be evaluated based on taxonomic utility. Apart from the primary principle of monophyly following Hennig (1966), Backlund and Bremer proposed secondary principles of classification such as maximizing stability, considering the support for monophyly, the ease of identification, and minimizing redundancy (i.e., maximizing phylogenetic information). These principles are generally followed by APG et al. (2016). Stevens [pers. comm. in Nickrent et al. (2010)] postulates two related principles: the preservation of groups well-established in literature and family size optimization. Additionally, Backlund and Bremer (1998) “…*believe that important phylogenetic information is best conveyed by names at the commonly used ranks of genus, family, order*….*”*

Here we present extensive phylogenetic tree inferences for relationships among the genera of Piperales, based on parsimony, likelihood and Bayesian inference (BI) methods, using data from all three subcellular genomes. We then test for potential phylogenomic discordance of inferences based on different genomic compartments, analyze and compare the topological results of the largest sampling of loci for Piperales to date, and conduct several topology tests to evaluate the recovered topologies. Finally, considering our phylogenomic results in perianth-bearing Piperales we discuss arguments for the reconsideration of their classification in light of the principles described by Hennig (1966), Backlund and Bremer (1998), and Smith et al. (2006).

## Materials and Methods

### Plant Material, DNA Extraction and Sequencing

Fresh leaf material of *Zippelia begoniifolia*, *Manekia incurva*, *Peperomia griseoargentea*, *Verhuellia lunaria*, *Anemopsis californica*, *Gymnotheca chinensis*, *Houttuynia cordata*, *Thottea sumatrana*, and *Saururus cernuus* was collected at the Botanical Garden in Dresden, Germany, cut into smaller fragments and dried in silica gel. Genomic DNA was extracted using the protocol of Doyle and Doyle (1987), modified to include an RNAse A (Thermo Scientific, Waltham, MA, United States) treatment (10 mg/ml). DNA concentration and quality were measured using a Qubit 3 Fluorometer (Thermofisher Scientific, Waltham, MA, United States) and Agilent Technologies 12-capillary Fragment Analyzer (Agilent, 2020) using the genomic DNA 50 kb kit. A paired-end (PE), 300 bp (base pairs) sequencing approach was carried out on a MiSeq (v.3, Illumina, San Diego, CA, United States) with 600 cycles. DNAs were sheared with an M220 ultrasonicator (Covaris, Inc., Woburn, MA, United States) to ∼600 bp and sequencing targeted about five million reads per sample. For *Thottea sumatrana*, ∼4 M. 150 bp PE reads were sequenced on an Illumina NextSeq500 platform with 500 bp insert size. Genome skimming data of *Lactoris fernandeziana* was created based on material used by Graham and Olmstead (2000). Library preparation and size selection followed methods described in Lam et al. (2015). The library was sequenced as 100 bp PE on a HiSeq platform (Illumina, San Diego, CA, United States) to produce ∼6 M. reads. Additionally, unpublished, assembled data for *Hydnora visseri* were provided by Naumann et al. (2016), for *Prosopanche americana* by Jost et al. (2020) and data for *Aristolochia fimbriata* were supplied by Yuannian Jiao (Chinese Academy of Sciences, China) as part of a yet unpublished paper.

### Data Mining From Public Repositories to Expand Sampling

Publicly available repositories such as GenBank (NCBI, 2020) and the sequence read archive (SRA, 2020) were mined for assembled organellar genomes or sequencing raw reads with the aim of retrieving data for missing ingroup genera. Additionally, data for one representative for each of several outgroup orders (Amborellales, Nymphaeales, Austrobaileyales, Chloranthales, Magnoliales, Laurales, and Canellales) were extracted to finalize the taxon sampling (Supplementary Table 1). Due to the non-availability of data for all three subcellular genomes for a single accession in Canellales, the data of *Drimys* (plastid and mitochondrial) and *Canella* (nuclear) were merged for the concatenated analyses. We are not trying to resolve phylogenetic relationships within the outgroup orders, therefore, this merging is not expected to have an impact on the ingroup results, given that the Canellales terminal serves to anchor that order.

### Raw Data Assembly and Extraction of Loci

Raw read data were assembled using the *de novo* assembly function in CLC Genomics Workbench (Qiagen, 2020), allowing for automatic calculation of optimal word and bubble sizes. Gene sequences of all three subcellular genomes for previously published taxa were filtered for the loci of interest (Supplementary Table 1). Assemblies were imported into Geneious v.11.1.5 (Geneious, 2020) and individually blasted (BLASTn, evalue 1e-10) for loci of interest from the plastid (pt) and mitochondrial (mt) genomes, using closely related reference species. 83 plastid genes were extracted, consisting of 79 protein coding genes and four ribosomal RNAs (rRNA), 44 mitochondrial genes (41 protein coding and three rRNAs). We also assembled a set of 13 nuclear (nc) loci that are expected to be single or low copy number based on studies of Duarte et al. (2010) and Jiao et al. (2011); those newly sequenced taxa were extracted using a dataset of cDNA sequences by Naumann et al. (2013), while the sampling was expanded with taxa that were obtained from multiple sources and accessions (Supplementary Table 1). We aimed for as few sampling gaps as possible; three of originally 13 nuclear loci were excluded from the analyses due to high amounts of missing data (i.e., <50% of sampled species represented).

### Phylogenetic Analyses

Single gene alignments were created in Geneious v.11.1.5 (Biomatters, Ltd., New Zealand) using the MAFFT alignment algorithm v.7.450 (Katoh et al., 2002; Katoh and Standley, 2013) and then manually checked and adjusted where necessary in AliView v.1.20 (Larsson, 2014). All genes belonging to the same genome were concatenated with SequenceMatrix v.1.8 (Vaidya et al., 2011), resulting in an 83-gene plastid matrix, a 44-gene mitochondrial matrix and a 10-gene nuclear matrix. A phylogeny based on a maximum likelihood (ML) analysis was created to check and verify that, when different data sources were employed for the same taxon, they were recovered as a monophyletic group when considering each source as a separate operational taxonomic unit (OTU) (Supplementary Figure 5 MSP), prior to merging them all into the same taxon. In addition to the 83-gene plastid matrix, a 21-gene plastid matrix was created, consisting only of the genes present in either of the two Hydnoraceae genera (Naumann et al., 2016; Jost et al., 2020).

Data were analyzed using parsimony, ML and BI approaches, both per genome and as concatenated sets of plastid, mitochondrial and nuclear data. Parsimony analysis was carried out using PAUP v.4.a165 (Swofford, 1998), implemented in Cipres Science Gateway (Miller et al., 2010) by using 1,000 heuristic searches and 1,000 bootstrap (BS) iterations, with the random starting tree option and the tree bisection-reconnection branch swapping method. Best fitting nucleotide substitution models for different ML analyses were estimated using jModelTest2 v. 2.1.6 (Darriba et al., 2012) and used as input for RAxML v.8.2.12 (Stamatakis, 2014), implemented in Cipres Science Gateway (Miller et al., 2010). ML analysis was carried out on complete data of concatenated gene sets of the individual genomes. In an attempt to reduce expected long branches leading to Hydnoraceae and to test their overall impact on the topology, we excluded the highly variable third codon position in specific analyses (for protein-coding genes only), and also inferred relationships based on amino acid alignments (protein-coding genes only, translated using Geneious v.11.1.5); although elevated mutational rates in parasitic plants are most apparent in the plastid genome, we repeated these variant analyses for all subcellular genomes, for consistency. The following different data partitioning approaches were also tested to accommodate different patterns of substitution in different subsets of the data: (1) by gene, (2) by gene and codon, (3) by assigning each 3rd codon position its own partition, and (4) unpartitioned (here referred to as single partition). Optimal partitioning schemes in each case were determined using PartitionFinder2 (Lanfear et al., 2014, 2017), and the respective output (i.e., partition combinations and their respective DNA substitution models) were used in the RAxML analyses. For the concatenated plastid, mitochondrial and nuclear data set (137 loci), ML trees were reconstructed using a single partition and a genome partition approach, as well as a translated (single partition) amino-acid sequence alignment. For all ML analyses, 1,000 bootstrap iterations were calculated. Finally, BI tree estimates for the fully concatenated unpartitioned and genome partitioned case were done using MrBayes v.3.2.7a (Huelsenbeck and Ronquist, 2001) on Cipres Science Gateway (Miller et al., 2010) with four chains and calculating 20 × 10^6^ generations, after which chains converged (assessed using the estimated sample size ESS) and a burn-in of 2 × 10^6^ was chosen. In addition to the above analyses, each complete single genome and concatenated nucleotide data set was considered with Hydnoraceae excluded (gene/genome partition, RAxML, 1,000 bootstrap iterations, Supplementary Figure 1), and using the genome partition approach we also performed a concatenated analysis of plastid and mitochondrial data only, including Hydnoraceae (RAxML, 1,000 bootstrap iterations, Supplementary Figure 6). Lastly, for the nuclear data set a coalescent tree was estimated using ASTRAL v.5.6.3 (Mirarab and Warnow, 2015; Sayyari and Mirarab, 2016), based on single gene trees. All trees were visualized using TreeGraph 2 (Stöver and Müller, 2010), with Amborellales defined as the outgroup. Taxon names in the phylogenetic trees are represented with either a binominal or genus only, depending on whether different accessions for a single genus were merged (in the latter case, sometimes different species, see above) to achieve the best locus-level coverage (Supplementary Table 1).

### Topology Testing

**FIGURE 4:**
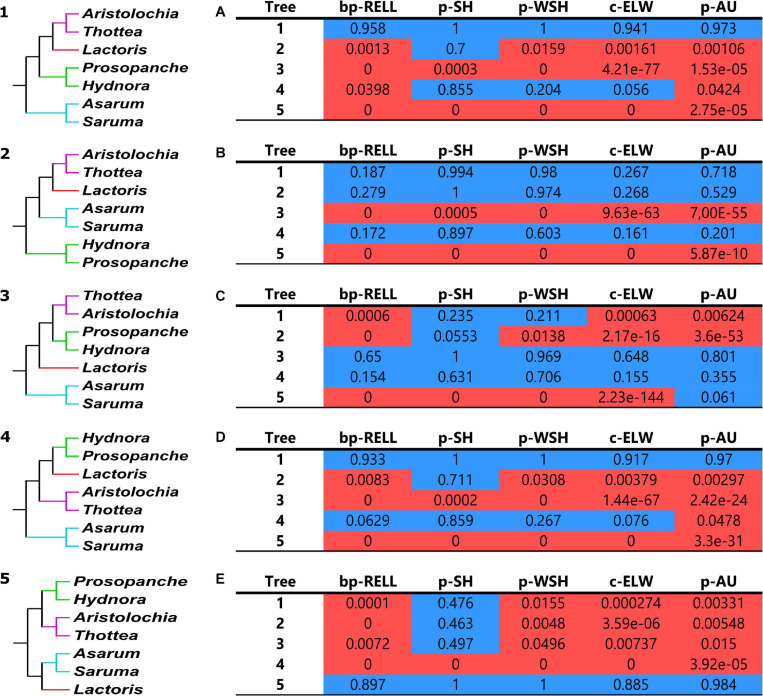
Topology test results for recovered topologies. Shown are the tested topologies **(1–5)** and tables **(A–E)** containing results of the bp-RELL, p-SH, p-WSH, c-ELW, and p-AU analyses. Tree numbers in the tables correspond to the topologies on the left **(1–5)**. Topology **(1)** contains clade II and was recovered for the concatenated three-genome data set (ML, genome partition), topology **(2)** was inferred for the 83 gene plastid data set (ML, assigning each 3rd codon position its own partition). Topology **(3)**, containing clade III, was reconstructed using the 44 gene mitochondrial data set (ML, gene partition), topology **(4)** using the concatenated 137-loci data set (MP, single partition), and topology **(5)** using the concatenated nuclear loci (ML, single partition). Table **(A)** shows the topology test results with topology **(1)** as null hypothesis, table **(B)** with topology **(2)** as null hypothesis and so on. Blue-colored values denote results within the 95% confidence sets; red-colored values denote significant exclusion. In the topologies **(1–5)**, perianth-bearing Piperales are color-coded: Asaraceae in turquoise, Aristolochiaceae in purple, Lactoridaceae in red and Hydnoraceae in green.

All 15 different, possible tree topologies for the four main lineages in the monophyletic perianth-bearing Piperales clade were tested for their significance using the tree topology evaluation tests implemented in IQ-Tree (Nguyen et al., 2015). Five of these topologies were recovered in one or more of our phylogenetic tree reconstructions. The tree files for the remaining ten topologies were manually created by altering only the relationships in the clade of interest. The bootstrap proportions using RELL (Kishino et al., 1990), SH test (Shimodaira and Hasegawa, 1999), weighted SH test, expected likelihood weight (ELW, Strimmer and Rambaut, 2002) and the approximately unbiased test (Shimodaira, 2002) were carried out in multiple runs. All tests performed 10,000 resamplings using the RELL method. We carried out five independent runs, one for each different topology recovered in our analyses. The program was provided with both the alignment file and substitution model used to infer the best tree for that data set (null hypothesis), as well as the set of alternative hypotheses (tree file containing all 15 possible topologies). For example, run A (Figure 4A) was provided with the data set reconstructing topology 1, and run B (Figure 4B) was provided with the data set underlying topology 2; significance was evaluated considering all 15 topologies.

## Results

### Dataset Characteristics

Assembly of newly generated next generation sequencing (NGS) data and database-mined loci of interest recovered varying amounts of data per accession and genome (Table 1 and Supplementary Table 1). 83 plastid loci were recovered for nearly all taxa sampled, with the exception of Hydnoraceae whose two genera have plastomes greatly reduced in gene content: here, only 20 plastid genes could be used for phylogenetic tree reconstruction. A total of 44 mitochondrial markers were recovered for almost all newly sequenced accessions, but fewer loci were retrieved for certain taxa sampled from GenBank (e.g., less than 50% of the total mt loci could be mined for *Asarum*, *Chloranthus*, *Drimys*, and *Saruma*). With regard to the recovered number of loci and the overall locus coverage, the nuclear data set was the most variable (Supplementary Figure 7). Complete coverage of all ten nuclear loci was achieved for only three accessions (*Aristolochia*, *Liriodendron*, and *Piper*), with only a single locus available for *Schisandra* and *Prosopanche* (Table 1 and Supplementary Figure 7).

**TABLE 1:** Overview of the number of character sets (charsets) and total sequence length (bp) recovered for the three subcellular genomes for all individual accessions represented in the sampling (see Supplementary Table 1 for more details).

**Taxon**	**Plastid**		**Mitochondrial**		**Nuclear**	
	**No. of charsets**	** Total length (bp)**	**No. of charsets**	** Total length (bp)**	**No. of charsets**	**Total length (bp)**
*Amborella*	83	86,218	39	45,796	9	5,186 bp
*Anemopsis*	83	86,222	43	46,972	8	2,143 bp
*Aristolochia*	83	86,222	44	50,050	10	6,422 bp
*Asarum*	82	85,466	20	22,110	6	2,716 bp
*Calycanthus*	83	86,195	44	45,399	9	4,764 bp
*Chloranthus*	83	86,211	10	19,230	6	3,981 bp
*Drimys/Canella*	83	86,212	11	19,714	9	2,176 bp
*Gymnotheca*	83	79,999	44	48,831	6	1,821 bp
*Houttuynia*	83	86,222	42	47,333	9	3,170 bp
*Hydnora*	20	34,324	42	47,062	9	5,192 bp
*Lactoris*	83	86,213	28	36,744	7	2,662 bp
*Liriodendron*	82	85,285	44	50,563	10	6,104 bp
*Manekia*	83	86,222	44	49,414	9	4,232 bp
*Nymphaea*	83	86,222	42	43,791	9	5,979 bp
*Peperomia*	83	86,222	41	41,551	5	2,500 bp
*Piper*	83	86,215	44	48,415	10	6,440 bp
*Prosopanche*	20	34,688	42	47,360	1	345 bp
*Saruma*	83	86,215	7	15,569	9	4,953 bp
*Saururus*	82	85,865	42	47,597	7	2,839 bp
*Schisandra*	83	86,195	44	50,233	1	482 bp
*Thottea*	83	86,222	44	50,010	9	3,374 bp
*Verhuellia*	83	86,222	44	50,446	8	3,161 bp
*Zippelia*	83	86,222	44	49,620	5	1,019 bp

*Accessions are ordered alphabetically. A maximum of 83 plastid loci, 44 mitochondrial loci, and 10 nuclear loci was aimed for. Taxa Drimys and Canella have been merged to achieve coverage for all three subcellular genomes for outgroup Canellales.*

### Molecular Phylogenomic Tree Reconstruction

#### Phylogenetic Tree Reconstructions Excluding Hydnoraceae

When Hydnoraceae are excluded from the datasets, virtually identical relationships are recovered across all analyses. Within the perianthless Piperales, Saururaceae, and Piperaceae are reconstructed as monophyletic and branch support values are very high (Supplementary Figure 1). In the latter family, *Manekia* + *Zippelia* is sister to *Peperomia* + *Piper*, and *Verhuellia* is sister to this entire clade. Within Saururaceae, the clades comprising *Gymnotheca* + *Saururus*, and *Anemopsis* + *Houttuynia*, have 100% support in all analyses, except in the analysis of nuclear data alone (Supplementary Figure 1). In the latter, *Anemopsis* is sister to the clade of *Gymnotheca* + *Saururus* with low support (BS 47%) and *Houttuynia* sister to the entire clade (BS 100%). Within perianth-bearing Piperales, relationships are identical between the plastid, mitochondrial, and concatenated data-based analyses (Supplementary Figure 1). Asaraceae are sister to the clade of Aristolochiaceae and Lactoridaceae with moderate to full support (BS 79–100%) and all families are monophyletic. Tree reconstruction based on the concatenated 10 nuclear loci recovered the clade Lactoridaceae and Asaraceae (BS 56%) sister to Aristolochiaceae + monophyletic perianthless Piperales (BS 83%, Supplementary Figure 1). Branch lengths within trees are relatively homogenous, with the shortest branches in Piperales recovered across the four data sets within Saururaceae, as well as within perianth-bearing Piperales and the branch leading to the latter (Supplementary Figure 1).

Hereafter, we only describe in detail the relationships within perianth-bearing Piperales; relationships within the perianth-less clade can be found, for each analysis, in the supporting material (Supplementary Figures 3–6). The topology within the latter clade is consistent across data sets, as well as types of analysis with very strong support, with the exception of some analyses based on nuclear data alone (Supplementary Figure 5).

**FIGURE 2:**
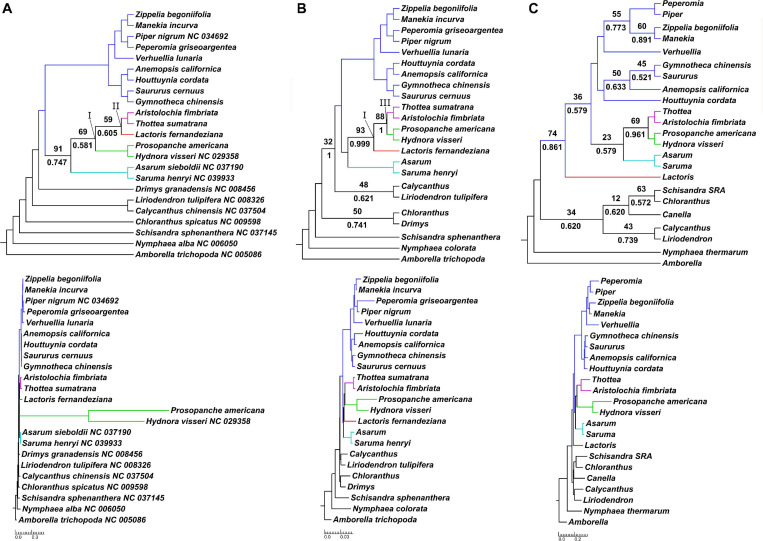
Phylogenomic discordance between plastid, mitochondrial and nuclear datasets. Results of maximum likelihood and Bayesian inference tree reconstruction based on gene-partitioned nucleotide data, as a cladogram with corresponding phylogram below, for **(A)** 83 plastid loci; **(B)** 44 mitochondrial loci; and **(C)** 10 nuclear loci. Support values are displayed for branches with <95% bootstrap support (above branch) or <0.95 posterior probability (below branch). Bootstrap support values are based on 1,000 pseudoreplicates. Piperales are color-coded: Asaraceae in turquoise, Aristolochiaceae in purple, Lactoridaceae in red and Hydnoraceae in green, Piperaceae and Saururaceae in blue. The three-family clade Aristolochiaceae, Hydnoraceae and Lactoridaceae is annotated with I, the clade Aristolochiaceae + Lactoridaceae with II, and the clade Aristolochiaceae + Hydnoraceae with III.

**FIGURE 3:**
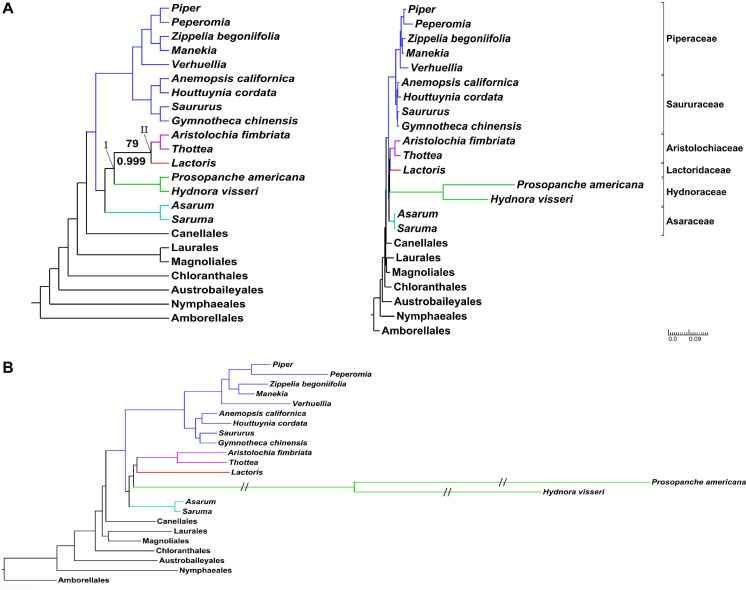
Piperales phylogeny based on genome-partitioned 137 loci of three subcellular genomes. **(A)** Topology with full support (BS 100%, PP 1) for all nodes, unless indicated for maximum likelihood analysis (bootstrap, above the branch) and Bayesian inference (posterior probability, below the branch). The right side shows the phylogram with annotated families of the Piperales. **(B)** Amplified view of branch lengths within Piperales. Branches annotated with “//” are shortened by 50%. Piperales are color-coded: Asaraceae in turquoise, Aristolochiaceae in purple, Lactoridaceae in red and Hydnoraceae in green, Piperaceae and Saururaceae in blue. The three-family clade Aristolochiaceae, Hydnoraceae and Lactoridaceae is annotated with I, the clade Aristolochiaceae + Lactoridaceae with II.

#### Phylogenetic Tree Reconstructions Including Hydnoraceae

Inclusion of Hydnoraceae leads to varying topologies depending on the subcellular origin of the data. Concatenated data or data with organellar origin typically recover two topologies for relationships within perianth-bearing Piperales, differing only in the relationships within the clade consisting of Aristolochiaceae, Hydnoraceae, and Lactoridaceae (Table 2 and Figures 2A,B). First, this three-family clade (referred to as clade I in Table 2) is well-supported in most analyses, although more weakly supported by the various analyses involving plastid data alone. Within this clade, a sub-clade comprising Aristolochiaceae and Lactoridaceae (referred to as clade II in Table 2), is recovered with poor to strong support for five out of seven analyses based on the concatenated organellar and concatenated three-genome data set (BS 24–79%, PP ∼0.99, Supplementary Figure 6 and Table 2) and with moderate support for all of the plastid-data derived inferences (BS 51–87%, PP 0.61–0.75; Figure 2A and Supplementary Figure 3); it tends to be less well-supported by partitioned data and is best supported in the 21-gene analysis of plastid data alone (BS 89%). The latter analysis includes only the plastid genes present in either of the Hydnoraceae genera. In contrast, a sub-clade comprising Aristolochiaceae and Hydnoraceae (referred to as clade III in Table 2 and Figure 2B) is recovered in all tree reconstructions based on mitochondrial data alone (ML, MP, and BI), with weak to strong support for this relationship (BS 56–88%, PP 0.96–1), and generally better support in partitioned likelihood analyses than the unpartitioned one. Clade III is also recovered in some inferences based on nuclear data alone (Figure 2C and Supplementary Figure 5), with low to moderate support (BS 23–75%, PP 0.96–0.97). Deletion of the third codon position appears to have little effect on support for either clade II or III for plastid or mitochondrial data (Table 2).

**TABLE 2:** Summary of bootstrap and posterior probability support for analyses supporting the two predominantly recovered topologies within perianth-bearing Piperales.

	**All combined**					**Organellar only**			**Plastid**							
	**137 SP**	**137 GnP**	**137 SP**	**137 GnP**		**127 SP**	**127 GnP**		**83 SP**	**83 GP**	**83 GCP**	**83 3SP**	**83 3GP**	**83 SP**	**83 GP**	**21 SP**

**Clade**	**ML**	**ML**	**BI**	**BI**		**ML**	**ML**		**ML**	**ML**	**ML**	**ML**	**ML**	**BI**	**BI**	**ML**
I	99	100	1	1		98	100		44	69	68	–	50	–	0.581	50
II	36	79	0.996	0.999		24	72		87	59	51	85	68	0.753	0.606	89

	**Mitochondrial**															
	**44 SP**	**44GP**	**44 GCP**	**44 3SP**	**44 3GP**	**44 ASP**	**44 SP**	**44 SP**	**44 GP**

	**ML**	**ML**	**ML**	**ML**	**ML**	**ML**	**MP**	**BI**	**BI**							

I	86	93	95	81	83	81	74	1	1							
III	64	88	84	56	81	57	62	0.964	1							

*Clade I refers to the clade comprising Aristolochiaceae, Hydnoraceae, and Lactoridaceae, clade II to the sister relationship of Aristolochiaceae and Lactoridaceae, and clade III to the sister relationship of Aristolochiaceae and Hydnoraceae. Analyses are displayed as number of loci and partitioning approach used, with single partition (SP), genome Partition (GnP), gene partition (GP), gene by codon partition (GCP), exclusion of the 3rd codon position (3SP, 3GP), and translated amino-acid data (ASP). Bootstrap support values are displayed for ML analyses and posterior probability for BI analyses. Dashes (–) highlight analyses without representation of a certain clade. Visual representation of the clades can be found in Figures 2, 3, as well as Supplementary Figures 3, 4, 6.*

#### Phylogenetic Tree Reconstructions Recovering Additional Topologies

Parsimony analysis of the concatenated 137-loci set recovers Lactoridaceae sister to Hydnoraceae, and Aristolochiaceae sister to that clade (Supplementary Figure 6). Tree reconstruction based on the amino-acid alignment of the same data set places Lactoridaceae sister to Aristolochiaceae + Hydnoraceae (Supplementary Figure 6), although with low support for the clade Aristolochiaceae + Hydnoraceae (BS 51%). Based on plastid data alone, Hydnoraceae were twice estimated to be sister to all remaining perianth-bearing Piperales although with poor support (BS 52–54%) (Supplementary Figure 3 ML, CP, and 3SP). A placement of Hydnoraceae close to the root of angiosperms was estimated for the translated amino-acid plastid data (Austrobaileyales sister to Hydnoraceae, Supplementary Figure 3 ASP) and MP analysis of the nucleotide data (Nymphaeales sister to Hydnoraceae, Supplementary Figure 3). Nuclear data-based tree reconstruction recovers Hydnoraceae sister to Aristolochiaceae in nine out of ten analyses (Figure 2C and Supplementary Figure 5) with weak to moderate support in ML analyses (BS 23–75%) and strong support in BI (PP 0.96–0.97). Asaraceae are placed sister to the aforementioned clade in multiple analyses (e.g., Figure 2C). Lactoridaceae placement is mostly ambiguous and poorly supported with either Lactoridaceae sister to Asaraceae (e.g., Supplementary Figure 5 ML and 10 SP) or sister to all other Piperales (e.g., Figure 2C). The inference based on coalescent analysis of the ten nuclear loci differed in some cases drastically from the concatenated one, based on the same input data set (Supplementary Figure 5). The coalescent analysis recovers paraphyletic perianth-bearing Piperales with Aristolochiaceae sister to the perianth-less clade and with Lactoridaceae sister to the clade Asaraceae + Hydnoraceae. Analyses with the third codon position excluded or based on an amino-acid alignment recover the paraphyly of perianth-bearing Piperales (Supplementary Figure 5), and the latter analysis also recovers the paraphyly of Hydnoraceae, although with poor support. Parsimony and BI recover a large polytomy, sometimes including multiple outgroup taxa (Supplementary Figure 5).

### Topology Testing

Within the perianth-bearing Piperales, a total of five discordant topologies with this clade monophyletic are recovered in this study and tested alongside the other 10 possible ones for their significance (Figure 4 and Supplementary Figure 2; there are 15 possible rooted arrangements of the four families, shown at the foot of Supplementary Figure 2, and note that the first five topologies in the latter are in the same order as the former figure). In summary, the first topology (Figures 3, 4.1) is recovered from the 137-loci combined analysis (organellar + nuclear data), using ML and partitioning by genome. The second topology (Supplementary Figure 3 ML 83, CP and Figure 4.2) was estimated using the 83 plastid data set and assigning the 3rd codon position its own partition. The topology reconstructed using the mitochondrial data set (ML analysis and gene partition, Figures 2B, 4.3) is the third topology tested. The fourth one is the result of the maximum parsimony analysis of the 137-loci data set (Supplementary Figure 6 and Figure 4.4), and the fifth topology was estimated for the ML analysis of the concatenated nuclear loci (single partition, Supplementary Figure 5 and Figure 4.5). In total, five independent analyses were run to test whether a specific data set rejects a certain topology. All runs were provided the identical set of topologies, corresponding to all possible topologies for a monophyletic perianth-bearing Piperales clade. The topologies differ only in the inferred relationships within the perianth-bearing Piperales (Figures 4.1–5). The runs themselves differed in the data set chosen as null hypothesis, e.g., run A (Figure 4A) was provided with the data set reconstructing topology 1, and run B (Figure 4B) was provided with the data set underlying topology 2.

All topologies performed best when the underlying data were set as null hypothesis, with the exception of topology 4, which performed only second best behind topology 1 (Figure 4D), although the null topology here was recovered using parsimony, not likelihood. Topology 1 (Hydnoraceae sister to the clade of Aristolochiaceae + Lactoridaceae) performed best in two out of five runs and was only significantly excluded twice (with exception of SH and WSH in run C and SH of run E). Both topology 2 (Hydnoraceae sister to all other perianth-bearing Piperales) and topology 3 (Lactoridaceae sister to the clade of Aristolochiaceae + Hydnoraceae) were significantly excluded in four out of five runs, except when their underlying data were set as the null hypothesis. Topology 4 (Aristolochiaceae sister to the clade of Hydnoraceae + Lactoridaceae) was only significantly excluded in the RELL and AU tests with topology 1 set as null hypothesis (Figure 4A), but was in no run the best performing one. Lastly, topology 5 (Lactoridaceae sister to Asaraceae and this clade sister to Aristolochiaceae + Hydnoraceae) was rejected by all analyses, except when it was set as the null hypothesis (Figure 4E). Run E also rejected all other tested topologies (with exception of the SH test).

The majority of the additional ten topologies (not recovered in this study; trees 6–10 in Supplementary Figure 2) were rejected by all tests in runs A, B, and D. One exception being topology 6 (Lactoridaceae + Aristolochiaceae sister to the clade of Asaraceae + Hydnoraceae) in run B (Supplementary Figure 2). Not rejected, but poorly performing are many of the additional topologies for run C, as well as the SH test of run E (Supplementary Figure 2). Topology 11 (Asaraceae sister to the clade of Aristolochiaceae + Hydnoraceae, with Lactoridaceae sister to this whole clade) is the only of the ten topologies rejected in only three out of five runs, yet not recovered in any of our inferences.

## Discussion

### Phylogenomic Discordance Among Genomic Compartments

Phylogenetic tree reconstructions of the magnoliid order Piperales at the genus level, excluding holoparasitic Hydnoraceae, recover the perianth-less Piperales clade; both inferences based on organellar data also recover a perianth-bearing clade (Supplementary Figure 1). The former comprises the two monophyletic families Piperaceae and Saururaceae. Within perianth-bearing Piperales, three clades are recovered, with Asaraceae sister to a clade of Lactoridaceae + Aristolochiaceae; these relationships received strong bootstrap support (BS 99–100%) for the concatenated and plastid data sets, as did those within perianthless Piperales based on mitochondrial data (BS 100%). Taxon bipartitions within perianth-bearing Piperales are well-supported based on the latter data. Nuclear-based phylogenies also recovered both Piperaceae and Saururaceae as monophyletic, although with lower support than in the aforementioned data sets, and perianth-bearing Piperales are recovered as non-monophyletic with weak support. The analyses based on nuclear single-copy locus data are potentially biased by the amount of missing data for several accessions (Table 1 and Supplementary Figure 7). Although this nuclear result could be based on cytonuclear discordance (e.g., shown in asterids, Stull et al., 2020) between Lactoridaceae and the other members of the perianth-bearing Piperales, we also cannot rule out the possibility that undiagnosed paralogy in subsets of the nuclear loci, particularly given the mixed sources of data for this subcellular genome (a combination of Sanger sequencing, some mined data without read information, and genome skimming with lower coverage for these loci). For example, in Rosaceae it has recently been shown, that for many “single copy” loci used in common target enrichment, paralogs can be found with increasing sequencing depth reflecting ancient gene duplication (Morales-Briones et al., 2020).

Extensive phylogenetic tree reconstructions that include the holoparasitic Hydnoraceae predominantly recover two topologies across data sets—differing only in the relationships within the clade comprising Aristolochiaceae, Hydnoraceae, and Lactoridaceae. The case with Hydnoraceae sister to Aristolochiaceae + Lactoridaceae (clade II) was generally well-supported for inferences based on loci from all three subcellular genomes combined (Figure 3), concatenated, organellar genomes only (Supplementary Figure 6, 127 OSP/OGnP) and plastid data alone (Figure 2A and Supplementary Figure 3). Support for this clade is highest when all data gathered were analyzed together, with moderate to strong support (both for ML and BI). The sister relationship of Hydnoraceae to the clade Lactoridaceae + Aristolochiaceae (e.g., Figure 3) is identical to the one recovered by Massoni et al. (2014), although here with the inclusion of *Prosopanche* and branches being well supported, for both the ML and BI analyses. Similar to previous studies (Massoni et al., 2014; Wanke et al., 2017), short branches, especially within perianth-bearing Piperales, are situated in close proximity to extremely long branches, not only leading to Hydnoraceae (Massoni et al., 2014), but also to the respective terminal branches for *Prosopanche* and *Hydnora* (Figure 3B; Jost et al., 2020). These drastic differences in branch lengths, together with the reduced number of available plastid markers, likely contributed to difficulties in previous studies that attempted to place these holoparasites. Analyses based on mitochondrial loci alone recover a different set of relationships (i.e., clade III instead of clade II; Figure 2 and Table 2). All inferences based on 44 mitochondrial loci recover Lactoridaceae as sister to the clade comprising Aristolochiaceae + Hydnoraceae, with low to strong support for ML, MP, and BI (Figure 2B and Supplementary Figure 4). In contrast to the plastid and concatenated results, branch lengths for the mitochondrial inferences are more homogenous across the tree, with short branches at the backbone in perianth-bearing Piperales but no drastic increase in Hydnoraceae. Phylogenomic discordance between the two organellar genomes is reflected not only by differences in topology, but also in branch lengths (rates of evolution), arising from a drastically reduced and rapidly evolving Hydnoraceae plastome (Naumann et al., 2016; Jost et al., 2020), in contrast to a mitochondrial genome that is presumably evolving at rates consistent with those of photosynthetic plants. Relationships inferred among outgroup orders also vary between different analyses based on mitochondrial data, which is most likely a result of the low number of loci derived from GenBank for some accessions (Table 1). The greatest differences with respect to number of loci and base pairs of sequence recovered are for the nuclear data, with *Prosopanche* and *Schisandra* represented by only a single locus (Table 1). These factors are most likely the reason for the various more unusual topologies recovered when reconstructing relationships using the nuclear data alone. Despite those differences, topologies within the perianth-less Piperales inferred based on the nuclear data are relatively stable, as are those within the perianth-bearing clade, with the placement of Lactoridaceae being the exception. Nonetheless, adding the nuclear data to the concatenated organellar data increases the support in comparison to the organellar data alone (Supplementary Figure 6).

The sister relationship of Lactoridaceae + Hydnoraceae, also inferred in the six-gene analysis of Nickrent et al. (2002), was recovered here in the MP analyses of our concatenated 137-loci data set (Supplementary Figure 6). Tree inferences in analyses that include plastid loci are potentially negatively affected by long branch attraction (LBA, Felsenstein, 1978; Hendy and Penny, 1989) when using a parsimony approach, and therefore might differ from inferences estimated using model-based methods (ML and BI). The latter phenomenon has previously been confirmed in, for example, holoparasitic Rafflesiales (Nickrent et al., 2004) and mycoheterotrophic plants (Lam et al., 2018). In our study, this is likely the case apparent when comparing likelihood results to the parsimony tree estimation of the mitochondrial data. Here, with mostly homogenous branch lengths across the mitochondrial tree, LBA is less likely to affect placement of taxa.

Overall, inferences based on mitochondrial data alone proved to be the most consistent across analyses with regards to topology. Topologies within Piperales were identical, regardless of analysis type (ML, MP, and BI), data reduction (3rd codon position excluded, translated amino-acid alignment) and partitioning approach. Inferences based on the concatenated three-genome data recovered an identical topology to the predominantly recovered one based on plastid data alone (clade II), though with much higher support for branches within perianth-bearing Piperales. Across all performed analyses, generally the use of gene partitioned ML analyses (genome partition for the concatenated analyses) tended to lead to the highest support values. Removing data subpartitions that are rapidly evolving (the 3rd codon position) or using amino-acid data and amino-acid substitution models (amino-acids evolve slower than nucleotide data) were unsuccessful in the sense that they yielded poorly supported trees with in some cases altered topology (Supplementary Figure 3 ASP); this may simply be a function of having too little data to make robust inferences in these cases.

### The Most Likely Phylogenetic Relationships Within Perianth-Bearing Piperales

Considering all the evidence, the most likely topology for relationships within perianth-bearing Piperales is the one recovered for the concatenated three genome analysis (Figure 3), with strong to full BS and PP support for Hydnoraceae sister to Lactoridaceae + Aristolochiaceae, and with Asaraceae sister to that clade. These results are identical to the poorly supported topology reported by Massoni et al. (2014), but here, with both genera of Hydnoraceae included and additional data considered per taxon, these relationships are well-supported. This topology receives additional support from the results of the conducted topology tests, evaluating the significance of all recovered relationships within perianth-bearing Piperales in comparison to one another, as well as in comparison to all other possible (but not recovered) topologies for the four families. The topology recovered by the six-gene analysis of Nickrent et al. (2002) is significantly excluded by the topology testing (in analysis 2 and 3), as well as the topology recovered for all analyses solely based on mitochondrial data (Figures 2B, 4.3, clade III), highlighting the discordance of genetic signals recovered for the two organellar genomes (Table 2). Within perianth-bearing Piperales, the uncertain placement may well be attributable to extremely short branches in close proximity to the extremely long ones that lead to Hydnoraceae. Missing plastid markers owing to plastome size reduction (Naumann et al., 2016; Jost et al., 2020), together with limited accessibility of plant material for Lactoridaceae, have made placement of Hydnoraceae difficult to infer in previous studies (Nickrent et al., 2010; Naumann et al., 2013; Massoni et al., 2014).

### Thoughts on Classification Within Perianth-Bearing Piperales

The classification of Piperales implemented by APG et al. (2016), prompted by the online survey of Christenhusz et al. (2015), needs reconsideration. Furthermore, discussions prompted by this survey are not only limited to this order. For example, Nyffeler and Eggli (2020) argued that the lumping done by APG within Asparagales “…*does not result in a gain of information*” and they argue to instead follow more traditional family circumscriptions until the proposed argument for higher practicability in Christenhusz et al. (2015) is proven. A similar argument was made by Nickrent (2020) against lumping of taxa in Santalales by APG et al. (2016). In Piperales, the lumping of Hydnoraceae and Lactoridaceae into Aristolochiaceae was based on two contradictory topologies available at that time (Naumann et al., 2013; Massoni et al., 2014). We argue that the problem of paraphyly in Aristolochiaceae s.l. (*Aristolochia*, *Asarum*, *Saruma*, and *Thottea*), also demonstrated in previous studies (Qiu et al., 2000; Soltis et al., 2000; Neinhuis et al., 2005; Wanke et al., 2007a,b) cannot simply be swept under the carpet by lumping Hydnoraceae and Lactoridaceae as well. A debate based on phylogenetic evidence, which we present here, has to be held, and the solutions that Smith et al. (2006) propose for such cases also have to be evaluated.

What are the alternatives and how do we decide among them? With the sound placement of Lactoridaceae and Hydnoraceae within Aristolochiaceae s.l., the latter could be recognized as a paraphyletic family, or split into multiple smaller ones, or the former two could be lumped into the family they are nested in, a broadly defined and monophyletic Aristolochiaceae. The first case (paraphyly) is generally undesirable, and the latter was recommended by Christenhusz et al. (2015) and implemented by APG et al. (2016). Lumping of the three families into Aristolochiaceae reduces Lactoridaceae and Hydnoraceae to subfamily status. While subfamilies Aristolochioideae Link, Asaroideae O. C. Schmidt and Hydnoroideae Walpers were previously described, they are rarely used. In addition, subfamily Lactoridoideae was not validly published by Christenhusz et al. (2015) according to the ICN (Art. 41.5, Turland et al., 2018), as the page number of the publication of its basionym Lactoridaceae was omitted (i.e., T.3 Abt.2: 19, Engler, 1887). To our knowledge this error has not been corrected elsewhere, and both Mabberley (2017) and Stevens (2001 onward) did not use this subfamily name and instead mentioned the genus name *Lactoris* along with the names of the other three subfamilies. There is no advantage in using subfamily over family names when both represent identical clades, especially if one of the subfamilies has to be newly introduced and an already established corresponding family name is available. Therefore, based on our data, we support the recognition of Hydnoraceae and Lactoridaceae, and a reversion to the earlier APG classifications (APG, 2003, 2009). We therefore accept four monophyletic families within the perianth-bearing Piperales, in line with Horner et al. (2015) and Nickrent (2020). Recognition of a narrowly defined Aristolochiaceae also requires recognition of Asaraceae, containing *Asarum* and *Saruma*, which are not closely related to Aristolochiaceae.

This classification with Asaraceae as a recognized family was also proposed by Nickrent (2020) who stated that this system within perianth-bearing Piperales “…*would result in the least amount of disruption*” and “…*would recognize the morphological distinctions among the members.*” The primary principle of monophyly of Hennig (1966) and the secondary principles of Backlund and Bremer (1998) are also met with our approach, which is maximizing stability, the support for monophyly, and minimizing redundancy. Additionally, each of the four distinct families within perianth-bearing Piperales are supported by clear apomorphies (see e.g., Stevens, 2001, onward), thus “*maximizing the ease of identification*” (Backlund and Bremer, 1998). The additional principle of preservation of groups well-established in the literature (Steven’s pers. comm. in Nickrent et al., 2010) is also met. As a service to society, a fundamental aspect of classification is its predictive quality (Stuessy, 2009a,b, 2013). The alternative approach of having broad classifications with fewer families places this aspect at risk, especially for lineages that are relatively unknown to many researchers and the general public (undoubtedly the case with Hydnoraceae and Lactoridaceae). We argue that a better approach is therefore to recognize multiple families of perianthless Piperales.

### Additional Considerations on the Families

Lactoridaceae, with its single remaining species *Lactoris fernandeziana*, are a relic of early angiosperm evolution (Stuessy et al., 1998) and are currently found only on a single island of the Juan Fernández Archipelago, Chile. Although the Juan Fernández Islands are relatively young volcanic islands (Stuessy et al., 1984; Ricci, 2001), fossil pollen of *Lactoripollenites* (=*Rosannia*) is widespread in the fossil record, from Late Cretaceous deposits from Namibia (Turonian-Campanian) to India, Australia, and North and South America (Zavada and Benson, 1987; Macphail et al., 1999; Gamerro and Barreda, 2008; Srivastava and Braman, 2010). Lactoridaceae are the only endemic angiosperm family of the Juan Fernández Islands and are an important signature plant for conservational efforts on the island flora. If Lactoridaceae were to lose their family status, this could impact the political acceptance of the conservational efforts (Stuessy et al., 2014). Ideally, political considerations must not influence taxonomic practice (Schmidt-Lebuhn, 2012); nonetheless, classification decisions may have political implications, particularly in conservation (Stuessy and Hörandl, 2014). When there is a choice, and good arguments can be made for recognizing such lineages as families, the answer seems clear: recognize the family.

In the past, genera *Hydnora* and *Prosopanche* have been relatively unknown to the botanical community as their occurrence is very local and rare. However, more recently their visibility has increased as new species are discovered and described (Bolin et al., 2011; Machado and Queiroz, 2012; Martel et al., 2018; Funez et al., 2019). If Lactoridaceae are recognized, as argued above, this in turn also supports recognition of Hydnoraceae (and Asaraceae) as distinct from the more narrowly defined Aristolochiaceae. This is supported by their rather bizarre morphology that is unique among angiosperms, and is consistent with the classification of other highly modified heterotrophic plants as families, such as Rafflesiaceae. The latter was accepted as a segregate family in Malpighiales by APG et al. (2016), in contrast to its inclusion in Euphorbiaceae s.l. by APG (2009), based on the same survey by Christenhusz et al. (2015), where a majority of respondents found it “…*difficult to conceive an expanded Euphorbiaceae that includes a taxon as divergent*.” Moreover, Hydnoraceae were not classified in Piperales until the study by Nickrent et al. (2002), which was then accepted by APG (2003). Prior to that, the family had generally been placed near Rafflesiaceae (e.g., Cronquist, 1988, who classified it in Rafflesiales, although recognizing Hydnoraceae as clearly distinctive).

Given the abovementioned arguments, we believe that the classification of perianth-bearing Piperales should therefore be reconsidered to recognize the four monophyletic families Aristolochiaceae, Asaraceae, Hydnoraceae, and Lactoridaceae.

## Data Availability Statement

The datasets presented in this study can be found in online repositories. The names of the repository/repositories and accession number(s) can be found below: TreeBase (http://purl.org/phylo/treebase/phylows/study/TB2:S27866).

## Author Contributions

SW: conception of the study. MJ, M-SS, IM, SG, and SW: data generation. MJ: analyses and visualization of results. MJ and SW: writing of the first draft. All authors reviewed and edited the draft and agreed to the published version of the manuscript.

## Conflict of Interest

The authors declare that the research was conducted in the absence of any commercial or financial relationships that could be construed as a potential conflict of interest.
