# Supplementary material for: Discordant Phylogenomic Placement of Hydnoraceae and Lactoridaceae Within Piperales Using Data From All Three Genomes
Source: Front Plant Sci. 2021 Apr 12;12:642598. doi: 10.3389/fpls.2021.642598 (PMC8072514; doi:10.3389/fpls.2021.642598)
Supplement: Supplementary file 1 [file Data_Sheet_1.docx]

# References for GenBank and SRA (sequence read archive) accessions listed in supplementary Table 1

Barkman, Todd J., Gordon Chenery, Joel R. McNeal, James Lyons-Weiler, Wayne J. Ellisens, Gerry Moore, Andrea D. Wolfe, and Claude W. DePamphilis. “Independent and Combined Analyses of Sequences from All Three Genomic Compartments Converge on the Root of Flowering Plant Phylogeny.” *Proceedings of the National Academy of Sciences* 97, no. 24 (2000): 13166–13171.

Bergthorsson, Ulfar, Aaron O. Richardson, Gregory J. Young, Leslie R. Goertzen, and Jeffrey D. Palmer. “Massive Horizontal Transfer of Mitochondrial Genes from Diverse Land Plant Donors to the Basal Angiosperm Amborella.” *Proceedings of the National Academy of Sciences* 101, no. 51 (2004): 17747–17752.

Cai, Zhengqiu, Cynthia Penaflor, Jennifer V. Kuehl, James Leebens-Mack, John E. Carlson, W. dePamphilis Claude, Jeffrey L. Boore, and Robert K. Jansen. “Complete Plastid Genome Sequences of Drimys, Liriodendron, and Piper: Implications for the Phylogenetic Relationships of Magnoliids.” *BMC Evolutionary Biology* 6, no. 1 (2006): 77.

Chen, Xiaodan, Jia Yang, Hao Zhang, Ru Bai, Xiao Zhang, Guoqing Bai, Panfeng Dai, and Guifang Zhao. “The Complete Chloroplast Genome of Calycanthus Chinensis, an Endangered Species Endemic to China.” *Conservation Genetics Resources* 11, no. 1 (2019): 55–58.

Davis, Jerrold I., Gitte Petersen, Ole Seberg, Dennis W. Stevenson, Christopher R. Hardy, Mark P. Simmons, Fabian A. Michelangeli, Douglas H. Goldman, Lisa M. Campbell, and Chelsea D. Specht. “Are Mitochondrial Genes Useful for the Analysis of Monocot Relationships?” *Taxon* 55, no. 4 (2006): 857–870.

Dong, Shanshan, Chaoxian Zhao, Fei Chen, Yanhui Liu, Shouzhou Zhang, Hong Wu, Liangsheng Zhang, and Yang Liu. “The Complete Mitochondrial Genome of the Early Flowering Plant Nymphaea Colorata Is Highly Repetitive with Low Recombination.” *BMC Genomics* 19, no. 1 (2018): 1–12.

Duarte, Jill M., P. Kerr Wall, Patrick P. Edger, Lena L. Landherr, Hong Ma, P. Kerr Pires, Jim Leebens-Mack, and W. dePamphilis Claude. “Identification of Shared Single Copy Nuclear Genes in Arabidopsis, Populus, Vitis and Oryzaand Their Phylogenetic Utility across Various Taxonomic Levels.” *BMC Evolutionary Biology* 10, no. 1 (2010): 61.

Goremykin, Vadim V., Karen I. Hirsch-Ernst, Stefan Wölfl, and Frank H. Hellwig. “Analysis of the Amborella Trichopoda Chloroplast Genome Sequence Suggests That Amborella Is Not a Basal Angiosperm.” *Molecular Biology and Evolution* 20, no. 9 (2003): 1499–1505.

———. “The Chloroplast Genome of Nymphaea Alba: Whole-Genome Analyses and the Problem of Identifying the Most Basal Angiosperm.” *Molecular Biology and Evolution* 21, no. 7 (2004): 1445–1454.

Hansen, Debra R., Sayantani G. Dastidar, Zhengqiu Cai, Cynthia Penaflor, Jennifer V. Kuehl, Jeffrey L. Boore, and Robert K. Jansen. “Phylogenetic and Evolutionary Implications of Complete Chloroplast Genome Sequences of Four Early-Diverging Angiosperms: Buxus (Buxaceae), Chloranthus (Chloranthaceae), Dioscorea (Dioscoreaceae), and Illicium (Schisandraceae).” *Molecular Phylogenetics and Evolution* 45, no. 2 (2007): 547–563.

Hu, Lisong, Zhongping Xu, Maojun Wang, Rui Fan, Daojun Yuan, Baoduo Wu, Huasong Wu, et al. “The Chromosome-Scale Reference Genome of Black Pepper Provides Insight into Piperine Biosynthesis.” *Nature Communications* 10, no. 1 (October 16, 2019): 4702. https://doi.org/10.1038/s41467-019-12607-6.

Jiao, Yuannian, Norman J. Wickett, Saravanaraj Ayyampalayam, André S. Chanderbali, Lena Landherr, Paula E. Ralph, Lynn P. Tomsho, Yi Hu, Haiying Liang, and Pamela S. Soltis. “Ancestral Polyploidy in Seed Plants and Angiosperms.” *Nature* 473, no. 7345 (2011): 97–100.

Jost, Matthias, Julia Naumann, Nicolás Rocamundi, Andrea A. Cocucci, and Stefan Wanke. “The First Plastid Genome of the Holoparasitic Genus Prosopanche (Hydnoraceae).” *Plants* 9, no. 3 (March 2020): 306. https://doi.org/10.3390/plants9030306.

KEW, Royal Botanical Gardens. “Data for the Release 0.1 of the Kew Tree of Life,” 2020.

Lim, Chae Eun, Sang-Choon Lee, Soonku So, Su-Min Han, Ji-Eun Choi, and Byoung-Yoon Lee. “The Complete Chloroplast Genome Sequence of Asarum Sieboldii Miq. (Aristolochiaceae), a Medicinal Plant in Korea,” 2018.

Lim, Jun Y., Charles R. Marshall, Elizabeth A. Zimmer, and Warren L. Wagner. “Multiple Colonizations of the Pacific by Peperomia (Piperaceae): Complex Patterns of Long-Distance Dispersal and Parallel Radiations on the Hawaiian Islands.” *Journal of Biogeography* 46, no. 12 (2019): 2651–2662.

Naumann, Julia, Joshua P. Der, Eric K. Wafula, Samuel S. Jones, Sarah T. Wagner, Loren A. Honaas, Paula E. Ralph, et al. “Detecting and Characterizing the Highly Divergent Plastid Genome of the Nonphotosynthetic Parasitic Plant Hydnora Visseri (Hydnoraceae).” *Genome Biology and Evolution* 8, no. 2 (February 1, 2016): 345–63. https://doi.org/10.1093/gbe/evv256.

Naumann, Julia, Karsten Salomo, Joshua P. Der, Eric K. Wafula, Jay F. Bolin, Erika Maass, Lena Frenzke, Marie-Stéphanie Samain, Christoph Neinhuis, and Claude W. dePamphilis. “Single-Copy Nuclear Genes Place Haustorial Hydnoraceae within Piperales and Reveal a Cretaceous Origin of Multiple Parasitic Angiosperm Lineages.” *PLoS One* 8, no. 11 (2013): e79204.

Parkinson, Christopher L., Keith L. Adams, and Jeffrey D. Palmer. “Multigene Analyses Identify the Three Earliest Lineages of Extant Flowering Plants.” *Current Biology* 9, no. 24 (1999): 1485–1491.

Petersen, Gitte, Ole Seberg, Argelia Cuenca, Dennis W. Stevenson, Marcela Thadeo, Jerrold I. Davis, Sean Graham, and T. Gregory Ross. “Phylogeny of the Alismatales (Monocotyledons) and the Relationship of A Corus (A Corales?).” *Cladistics* 32, no. 2 (2016): 141–159.

Povilus, Rebecca A., Jeffrey M. DaCosta, Christopher Grassa, Prasad RV Satyaki, Morgan Moeglein, Johan Jaenisch, Zhenxiang Xi, Sarah Mathews, Mary Gehring, and Charles C. Davis. “Water Lily (Nymphaea Thermarum) Genome Reveals Variable Genomic Signatures of Ancient Vascular Cambium Losses.” *Proceedings of the National Academy of Sciences* 117, no. 15 (2020): 8649–8656.

Qiu, Yin-Long, Olena Dombrovska, Jungho Lee, Libo Li, Barbara A. Whitlock, Fabiana Bernasconi-Quadroni, Joshua S. Rest, Charles C. Davis, Thomas Borsch, and Khidir W. Hilu. “Phylogenetic Analyses of Basal Angiosperms Based on Nine Plastid, Mitochondrial, and Nuclear Genes.” *International Journal of Plant Sciences* 166, no. 5 (2005): 815–842.

Qiu, Yin-Long, Jungho Lee, Fabiana Bernasconi-Quadroni, Douglas E. Soltis, Pamela S. Soltis, Michael Zanis, Elizabeth A. Zimmer, Zhiduan Chen, Vincent Savolainen, and Mark W. Chase. “The Earliest Angiosperms: Evidence from Mitochondrial, Plastid and Nuclear Genomes.” *Nature* 402, no. 6760 (1999): 404–407.

Qiu, Yin-Long, Libo Li, Tory A. Hendry, Ruiqi Li, David W. Taylor, Michael J. Issa, Alexander J. Ronen, Mona L. Vekaria, and Adam M. White. “Reconstructing the Basal Angiosperm Phylogeny: Evaluating Information Content of Mitochondrial Genes.” *Taxon* 55, no. 4 (2006): 837–856.

Qiu, Yin-Long, Libo Li, Bin Wang, Jia-Yu XUE, Tory A. Hendry, Rui-Qi LI, Joseph W. Brown, Yang Liu, Geordan T. Hudson, and Zhi-Duan CHEN. “Angiosperm Phylogeny Inferred from Sequences of Four Mitochondrial Genes.” *Journal of Systematics and Evolution* 48, no. 6 (2010): 391–425.

Rice, Danny W., Andrew J. Alverson, Aaron O. Richardson, Gregory J. Young, M. Virginia Sanchez-Puerta, Jérôme Munzinger, Kerrie Barry, Jeffrey L. Boore, Yan Zhang, and Claude W. dePamphilis. “Horizontal Transfer of Entire Genomes via Mitochondrial Fusion in the Angiosperm Amborella.” *Science* 342, no. 6165 (2013): 1468–1473.

Richardson, Aaron O., Danny W. Rice, Gregory J. Young, Andrew J. Alverson, and Jeffrey D. Palmer. “The ‘Fossilized’ Mitochondrial Genome of Liriodendron Tulipifera: Ancestral Gene Content and Order, Ancestral Editing Sites, and Extraordinarily Low Mutation Rate.” *BMC Biology* 11, no. 1 (2013): 1–17.

Shang, Junzhong, Jingpu Tian, Huihui Cheng, Qiaomu Yan, Lai Li, Abbas Jamal, Zhongping Xu, et al. “The Chromosome-Level Wintersweet (Chimonanthus Praecox) Genome Provides Insights into Floral Scent Biosynthesis and Flowering in Winter.” *Genome Biology* 21, no. 1 (August 10, 2020): 200. https://doi.org/10.1186/s13059-020-02088-y.

Sinn, Brandon T., Dylan D. Sedmak, Lawrence M. Kelly, and John V. Freudenstein. “Total Duplication of the Small Single Copy Region in the Angiosperm Plastome: Rearrangement and Inverted Repeat Instability in Asarum.” *American Journal of Botany* 105, no. 1 (2018): 71–84. https://doi.org/10.1002/ajb2.1001.

Yu, X., Y. Feng, W. Zhai, M. Chen, and G. Wu. “The Complete Mitochondrial Genome of Schisandra Sphenanthera            (Schisandraceae).” *Unpublished*, 2019.

Yu, X.X., Y.L. Feng, Y.Z. Shao, and G.X. Wu. “The Complete Chloroplast Genome Sequence of Schisandra Sphenanthera.” *Unpublished*, 2018.

Zhang, N., S.M. Handy, and J. Wen. “Plastid Genomes of Illicium,” 2017.
