# Supplementary figures and images for: Discordant Phylogenomic Placement of Hydnoraceae and Lactoridaceae Within Piperales Using Data From All Three Genomes
Source: Front Plant Sci. 2021 Apr 12;12:642598. doi: 10.3389/fpls.2021.642598 (PMC8072514; doi:10.3389/fpls.2021.642598)

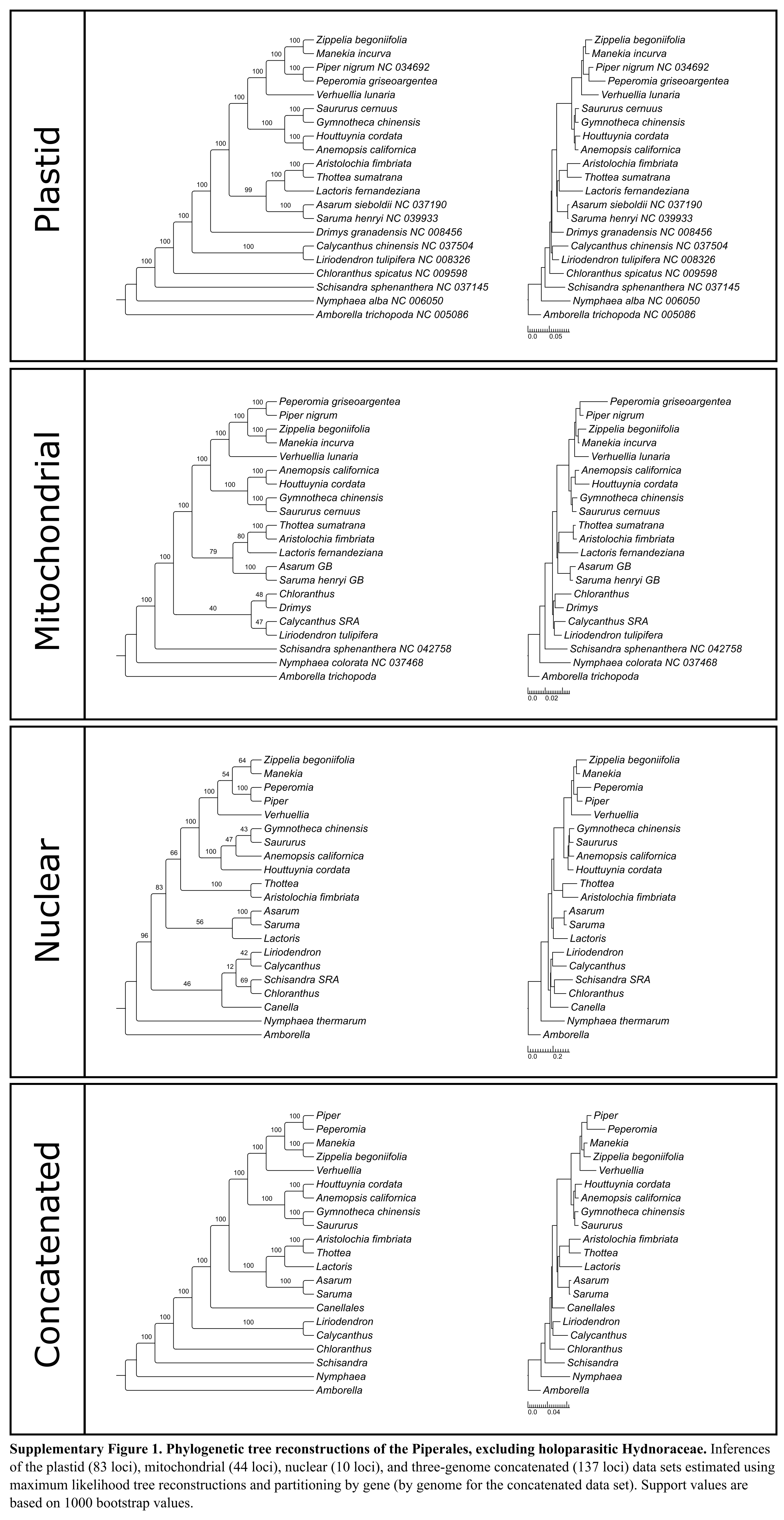

Supplement: Supplementary file 3 [file Image_1.JPEG]

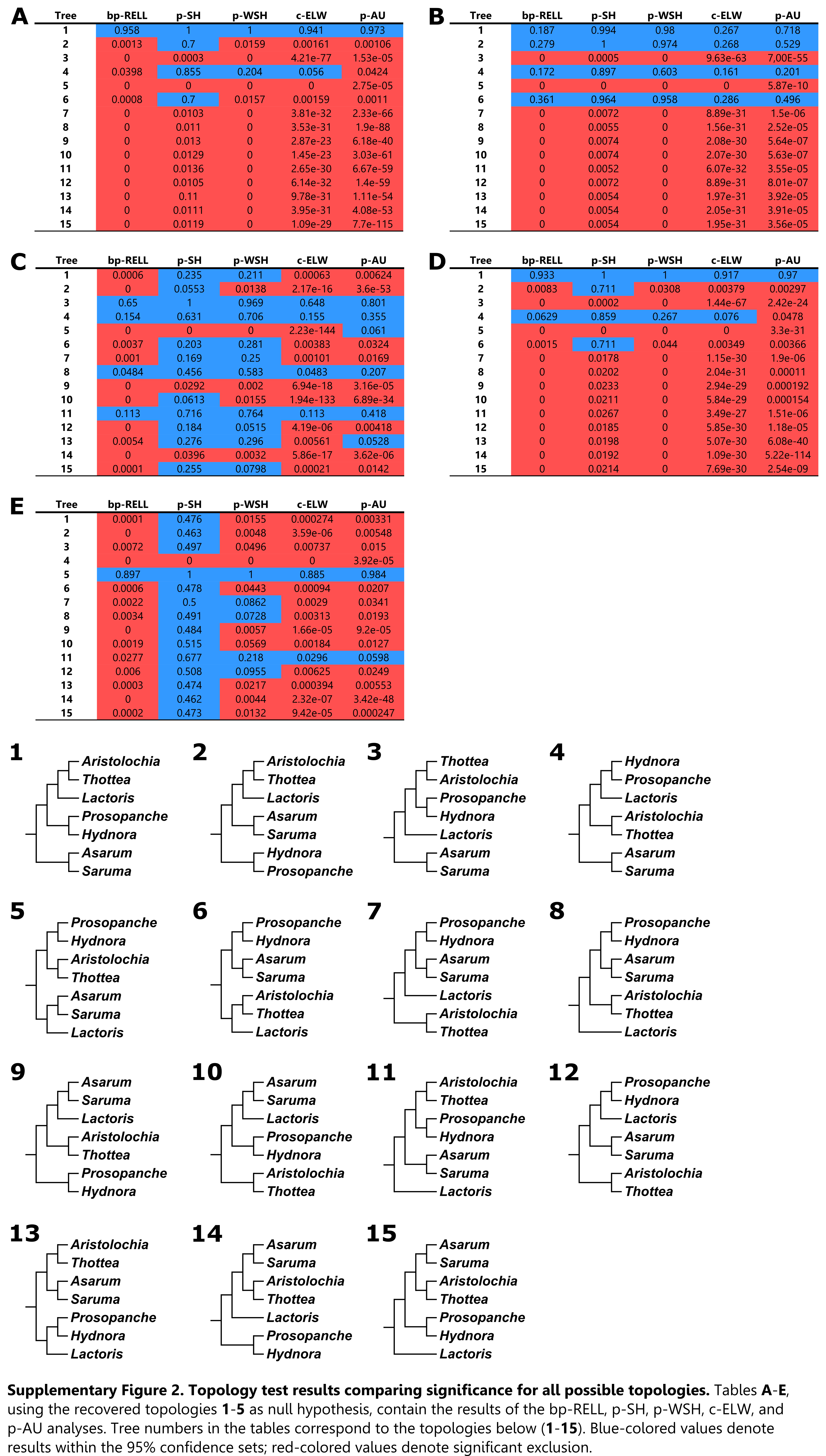

Supplement: Supplementary file 4 [file Image_2.JPEG]

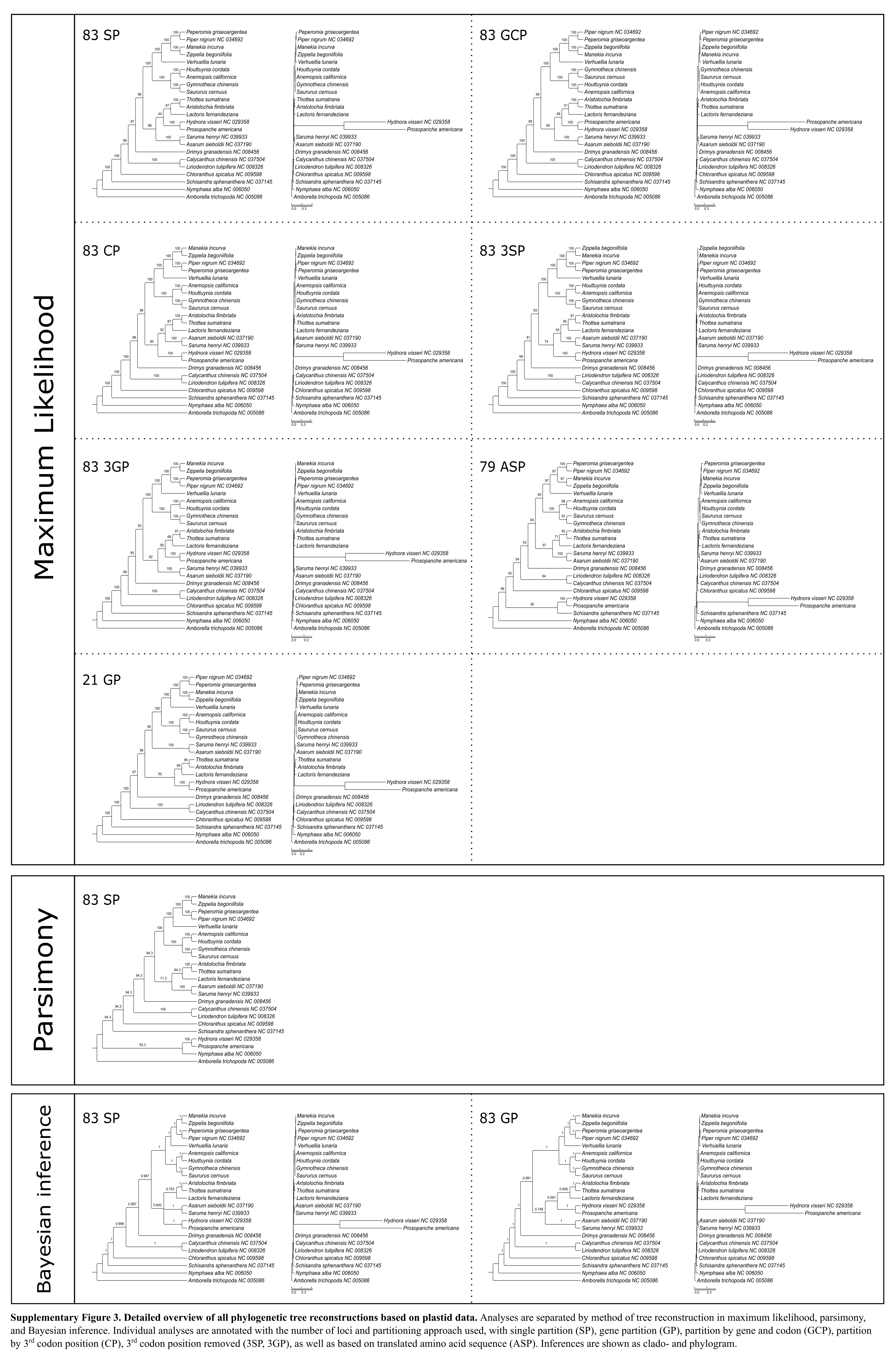

Supplement: Supplementary file 5 [file Image_3.JPEG]

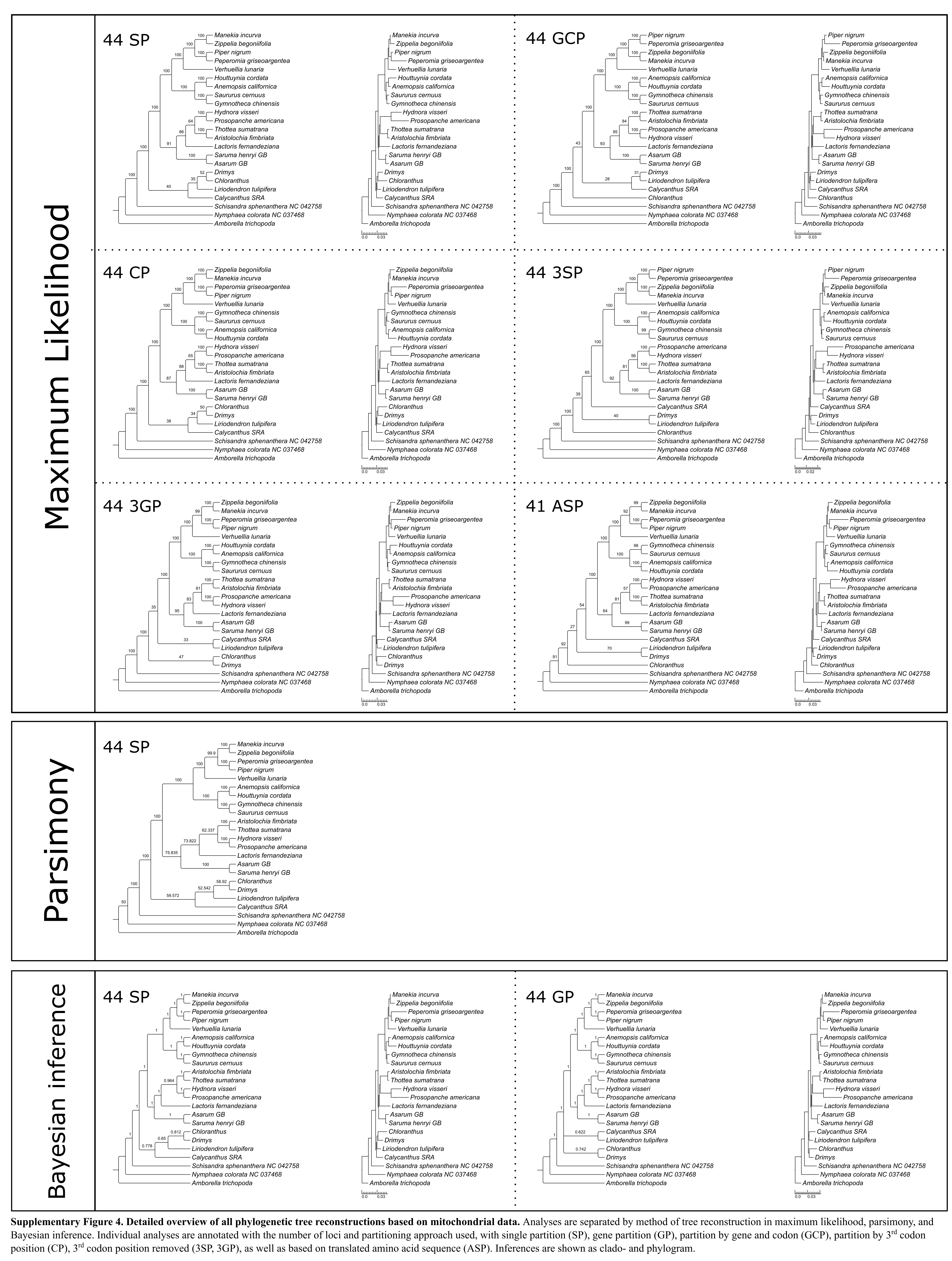

Supplement: Supplementary file 6 [file Image_4.JPEG]

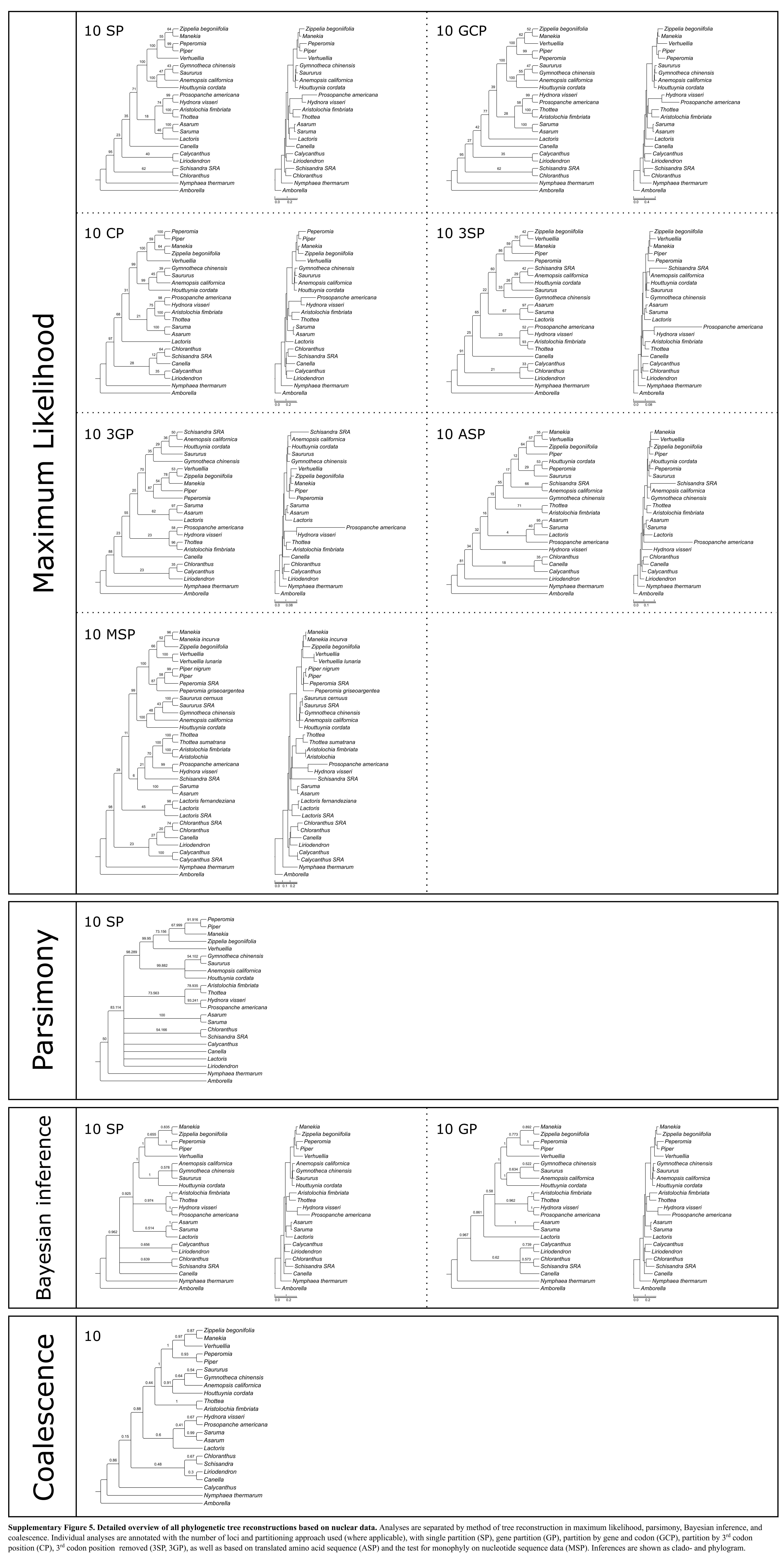

Supplement: Supplementary file 7 [file Image_5.JPEG]

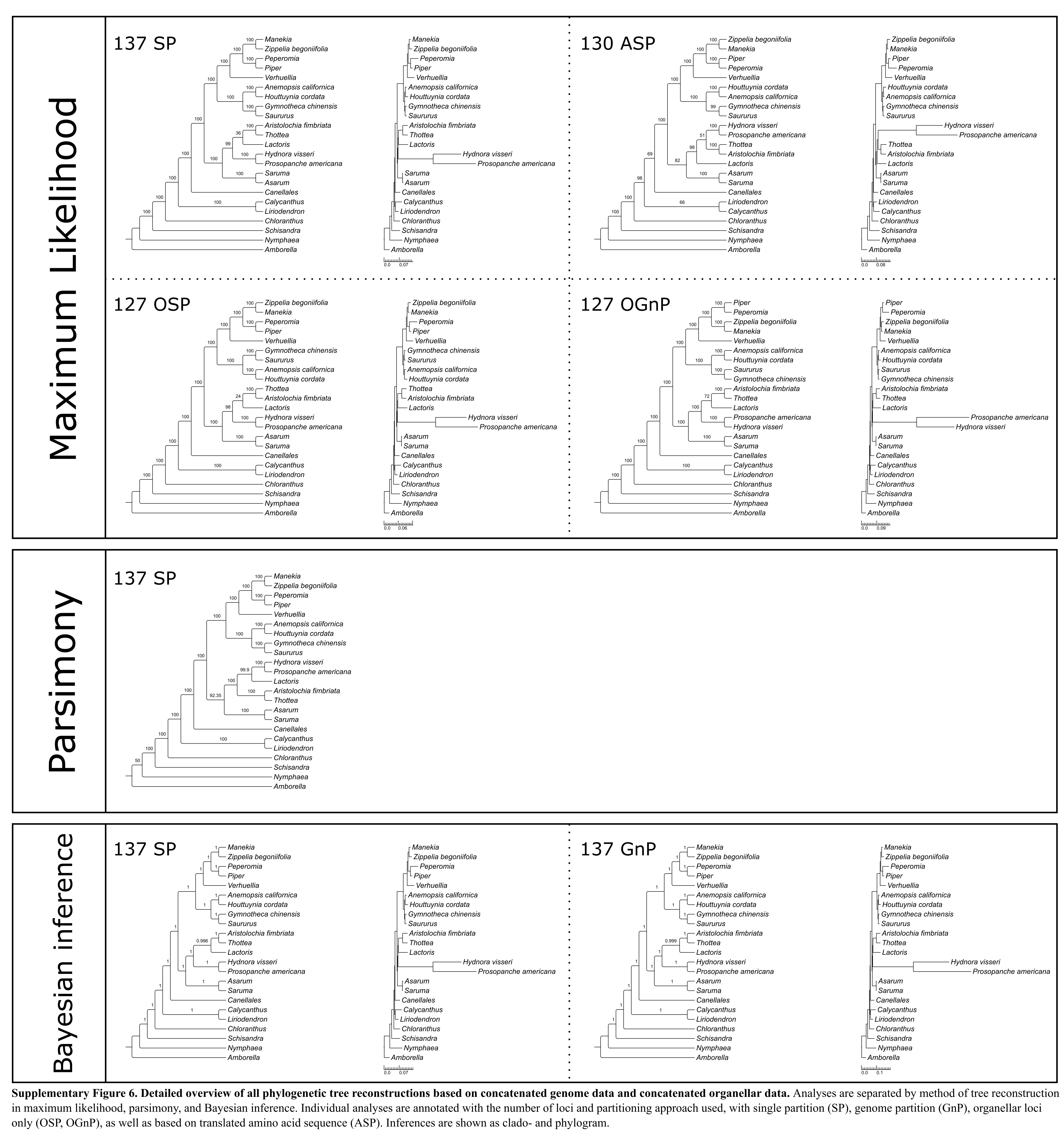

Supplement: Supplementary file 8 [file Image_6.JPEG]

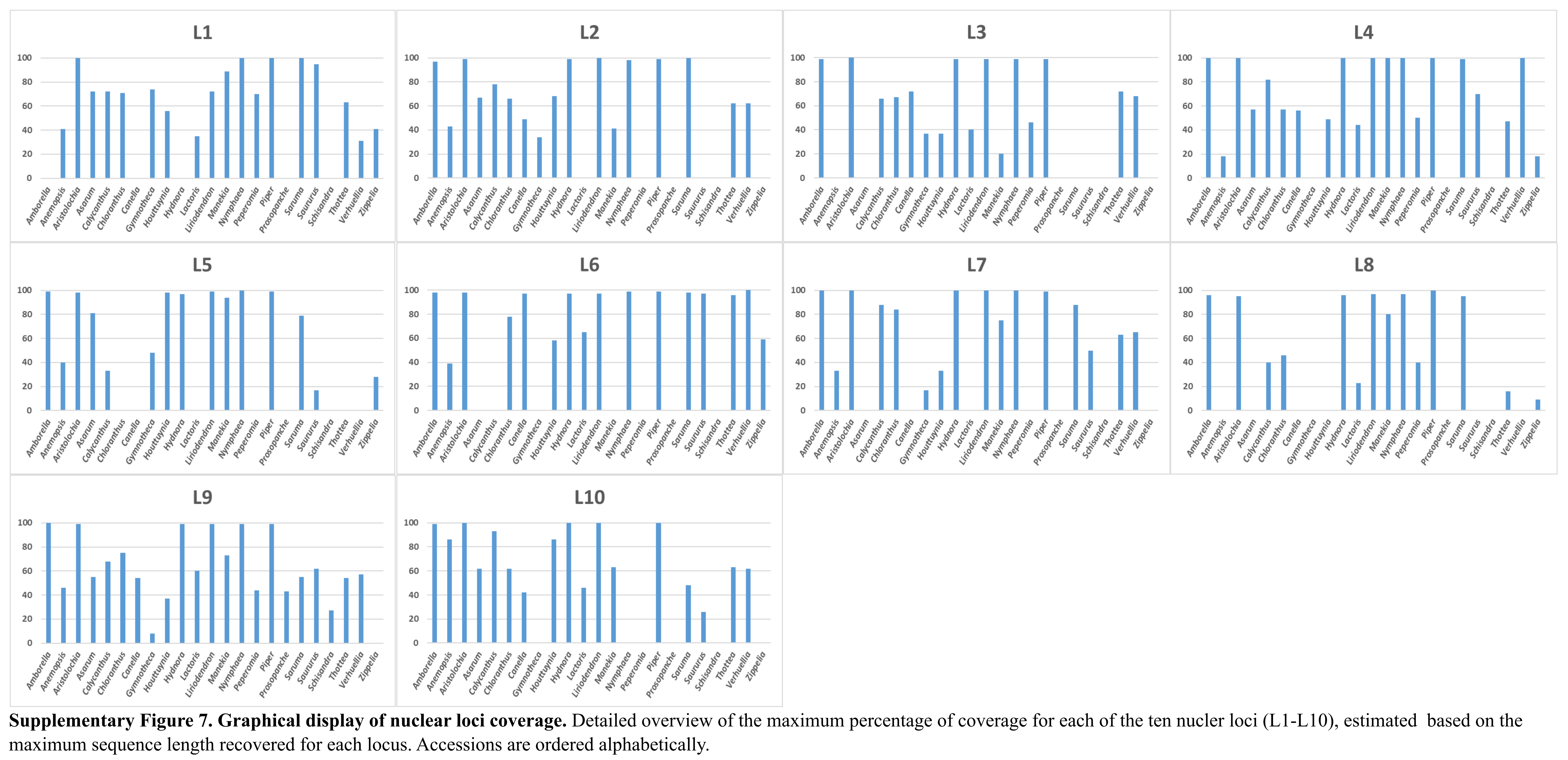

Supplement: Supplementary file 9 [file Image_7.JPEG]
